# Alterations of the Human Gut Microbiota in Intrahepatic Cholestasis of Pregnancy

**DOI:** 10.3389/fcimb.2021.635680

**Published:** 2021-04-30

**Authors:** Qitao Zhan, Xuchen Qi, Ruopeng Weng, Fangfang Xi, Yuan Chen, Yayun Wang, Wen Hu, Baihui Zhao, Qiong Luo

**Affiliations:** ^1^ Department of Obstetrics, Women’s Hospital, School of Medicine, Zhejiang University, Hangzhou, China; ^2^ Department of Neurosurgery, Sir Run Run Shaw Hospital, School of Medicine, Zhejiang University, Hangzhou, China

**Keywords:** severe ICP, *Escherichia_Shigella*, *Olsenella*, *Turicibacter*, biosynthesis of unsaturated fatty acids metabolism, propanoate metabolism pathway

## Abstract

**Background and Aims:**

Women with severe intrahepatic cholestasis of pregnancy (ICP) are at higher risks of fetal complications and without effective treatments. Changes in gut microbiota in pregnancy were found to be related to the altered intestinal bile acid composition, so we aimed to explore the alterations of microbiota in the gut of ICP patients.

**Methods:**

A total of 90 women were recruited, including 45 ICP patients and 45 healthy controls. The gut microbiota communities of ICP group were compared to control group through 16S ribosomal RNA gene sequencing. The results were then confirmed by real-time polymerase chain reaction (PCR) and generalized linear model (GLM). Furthermore, we analyzed the relationships between microbiota and the severity of ICP.

**Results:**

A total of seven genera and nine taxa with differential abundances between the ICP patients and the controls were identified. All of the seven genera were verified through real-time PCR, and three key genera *Parabacteroides*, *Flavonifractor*, and *Megamonas* were confirmed by using the GLM model. Further analysis found that the genera *Escherichia_Shigella*, *Olsenella*, and *Turicibacter* were enriched in the severe ICP group, the microbial gene function related to biosynthesis of unsaturated fatty acids and propanoate metabolism were also increased in them.

**Conclusions:**

Overall, our study was the first in Asia to demonstrate an association between gut microbiota and ICP. Our findings would contribute to a better understanding of the occurrence of ICP.

## Introduction

Intrahepatic cholestasis of pregnancy (ICP) is a pregnancy-specific and primary liver disease that typically presents in the second or third trimester. The clinical manifestations are maternal itching, jaundice, and persistently deranged liver function, including raised serum bile acids. The reported incidence of ICP is variable geographically from 0.2 to 2% (Kenyon et al., 2010; Wikström Shemer et al., 2013; Williamson and Geenes, 2014), but the recurrence rate is as high as 40–70% (Kenyon et al., 2010). In severe ICP patients, it was reported that the fetal complications would increase by 1–2% per additional 1 μmol/L of bile acid (Glantz et al., 2004). Compared with controls, severe ICP patients were reported with increased risks of preterm delivery, neonatal unit admission, and stillbirth (Ovadia et al., 2019b). Ursodeoxycholic acid (UDCA) is one of the secondary bile acids, which are metabolic byproducts of intestinal bacteria (Wahlström et al., 2016). UDCA or combined with s-adenosylmethionine (SAMe) is currently the first-line treatment for ICP. Although the drugs were considered effective to improve maternal itching, there is still insufficient evidence of improved perinatal outcomes (Chappell et al., 2018).

The liver interacts with the gut directly *via* hepatic portal and bile secretion systems. Sayin et al. showed that the gut microbiota regulated expressions of several enzymes, which were the rate-limiting enzymes and determined the amount of bile acids produced (Sayin et al., 2013). The process of deconjugation of bile acid was carried out by bacteria with bile salt hydrolase activity (Jones et al., 2008). Recent studies found that gut microbiota changed dramatically from first trimesters to third trimesters in pregnancy with an overall increase in Proteobacteria and Actinobacteria and reduced richness (Koren et al., 2012). Changes in gut microbiota in pregnancy were found to be related to the altered intestinal bile acid composition (Ovadia et al., 2019a). In ICP, the bile flow and intestinal bile acid contents might be affected, which in turn influence the gut microbiota (Pataia et al., 2020). UDCA treatment not only enriched bile salt hydrolase-expressing Bacteroidetes, which are essential for bile acid deconjugation (Ovadia et al., 2020), but also reduced the feto-maternal transplacental gradient (Ren L. et al., 2019). Animal study also found the effects of probiotic in the prevention of the bile acid disorders (Geenes et al., 2014). But the relationships between bile acid changes in ICP and gut microbiota have not been fully studied yet.

On the basis of that, we hypothesized that alterations in the gut microbiota might exist in ICP patients. We firstly compared the gut microbiota communities of ICP patients with healthy controls using 16S ribosomal RNA (16s rRNA) gene amplicon sequencing. Secondly, we used real-time polymerase chain reaction (PCR) to confirm the differentially abundant taxa between the ICP patients and healthy controls. Then, we found the key genera through the generalized linear model (GLM) after eliminating possible interferences. Furthermore, we analyzed the relationships among the microbiota, the severity of ICP, and the clinical characteristics of ICP patients to have a better understanding of the occurrence of ICP.

## Material and Methods

### Study Population and Sample Collection

Fecal samples were obtained from 90 pregnant women in Women’s Hospital, School of Medicine, Zhejiang University, between 1st April, 2018 and 1st December, 2019. The inclusion criteria of current study were as follows: 1) gestational week was greater than the 28th week (the third trimester); 2) without complications which might affect microbiome composition such as pregnancy diabetes, hypertensive disorders of pregnancy, thyroid disorders, and viral hepatitis; 3) had not received any antibiotic treatment 3 month before; 4) had not taken probiotics 2 weeks before sample collection. Of the 90 eligible women, 45 had a diagnosis of ICP were included as the experiment group, and the other 45 women of non-ICP were matched age and height as the control group. The pre-pregnancy body mass index in both groups was less than 28kg/m^2^ to exclude the possible influence of obesity on the gut microbiota. The ICP patients were all meet the criteria of cholyglycine (CG) greater than two times the upper limit of normal (normal range is 0–4.6 μmol/L) and total bile acid (TBA) greater than 10 μmol/L (normal range is 0–10 μmol/L). All symptoms and laboratory abnormalities of the ICP patients normalize after deliveries.

Fecal samples and all the blood samples were collected when the patients were fasting at least 8 h and before the patients were treated with UDCA or combined with SAMe. To ensure the freshness of samples, hospitalized ICP patients and randomly controlled healthy women who were not in labor were recruited. In order to reduce the impact of gestational week on the gut microbiota, the sampling gestational weeks of both groups were strictly matched within 1 week. Two of the authors were responsible for the collection, and the samples were collected immediately upon defecation, and then stored at −80°C freezers within 30 min after being collected. Pre-pregnancy characteristics, characteristics during delivery, and the levels of CG and TBA were summarized in **Table 1**. There were 10 severe (TBA exceed 40 μmol/L) and 35 mild (TBA range 10–39 μmol/L) ICP patients included.

**Table 1:** Characteristics of the subjects.

Characteristics		ICP (n = 45)	CON (n = 45)	*p* Value
Pre-pregnancy	Age (years)	30.0 ± 4.0	30.4 ± 4.2	0.646
	Height (m)	1.60 ± 0.05	1.61 ± 0.05	0.243
At delivery	WeightGain^‡^ (kg)	11.2 ± 2.8	12.9 ± 3.1	0.007
	Weight (kg)	63.7 ± 7.2	69.3 ± 7.8	0.001
	BMI^†^ (kg/m^2^)	24.9 ± 2.7	26.7 ± 2.4	0.002
	GWofBirth^*^ (weeks)	37 [36,38]	39 [38, 40]	<0.000
TBA^¶^ (μmol/L)		21.0 [13.5, 29.0]	2.0 [1.0, 3.0]	<0.000
CG^#^ (μmol/L)		15.76 [10.65, 23.89]	2.53 [1.99, 3.28]	<0.000

^‡^WeightGain, weight that gained during pregnancy; †BMI, body mass index; ^*^GWofBirth, gestational weeks of birth; ^¶^TBA, total bile acid; ^#^CG, cholyglycine.

### DNA Extraction, PCR Amplification, and Sequencing

DNA was extracted from 180 to 200 mg of fecal samples using a QIAamp Fast DNA Stool Mini extraction Kit (Qiagen, Germany) according to manufacturer’s protocols. The V3-V4 region of the bacteria 16S ribosomal RNA genes were amplified by PCR amplification using specific primers supplemented with Illumina sequencing adapters and sample-specific barcodes according to Illumina’s instructions (https://support.illumina.com/downloads/16s_metagenomic_sequencing_library_preparation.html). The V3-V4 regions of the bacterial 16S rRNA gene were amplified using the universal primers 341 F 5’ -barcode- CCTACGGGRSGCAGCAG)-3’ and 806 R 5’ -barcode- GGACTACVVGGGTATCTAATC-3’, which contain a short sequence barcode unique to each sample. Extracted DNA of each sample was kept frozen at −20°C (see **Supplemental Method 1.1** for detail). Amplicons were extracted from 2% agarose gels and purified using the AxyPrep DNA Gel Extraction Kit (Axygen Biosciences, Union City, CA, USA) according to the manufacturer’s instructions and quantified using Qubit^®^2.0 (Invitrogen, USA). After preparation of library, these tags were sequenced on MiSeq platform (Illumina, Inc., CA, USA) for paired end reads of 250 base pairs (bp).

### Process of Sequencing Data

OTU were clustered with 97% similarity using UPARSE (Edgar, 2013) (http://drive5.com/uparse/) and chimeric sequences were identified and removed using Usearch (version 7.0). Each representative tag was assigned to a taxa by Ribosomal Database Project (RDP) Classifier (http://rdp.cme.msu.edu/) against the RDP database (http://rdp.cme.msu.edu/) using confidence threshold of 0.8. OTU profiling table and α-/β- diversity analyses were also achieved by python scripts of Qiime (V1.9.1) (see **Supplemental Method 1.3** for detail). The total samples resulted in 1,077,564 clean reads with an average of 35,918.8 ± 1,980.3 clean tags per sample. The data was deposited in the NCBI database and our SRA record is accessible with the following link: https://www.ncbi.nlm.nih.gov/sra/PRJNA684451.

For taxa with a prevalence ≥10%, differential abundance analysis was performed using the Wilcoxon rank-sum test at the phylum, class, order, family, genus, and OTU levels to find out the phylotypes that had a significant effect on the division between groups. For multiple comparisons of bacterial counts, the false discovery rate was calculated using the Benjamini and Hochberg method. Microorganism features used to distinguish the microbiotas specific to ICP were identified using the linear discriminant analysis (LDA) effect size (LEfSe) method with an alpha cutoff of 0.05 and an effect size cutoff of 2.0.

A Phylogenetic Investigation of Communities by Reconstruction of Unobserved States (PICRUSt) test, based on the *de novo* OTUs through the RDP database (http://rdp.cme.msu.edu/), was used to predict the abundances of functional categories in the Kyoto Encyclopedia of Genes and Genomes (KEGG) orthologs (KO). The graph of KEGG pathways in level 2 (41 pathways) and level 3 (328 pathways) 2 was performed with STAMP, and *p* values were calculated with White’s non-parametric t-test.

### Genus-Specific Quantification by Real-Time PCR

The comparison and analysis of the 16S region sequences with those of differential genera reference strain were carried out by the multiple alignment programs DNAman software. The specific primer target sites for the quantitative analysis were performed using Primer 5. Universal 16S rRNA gene was used as the internal reference and the abundances of the genera were expressed as relative levels to 16S rRNA. The PCR reaction and condition were the same with 16S rRNA gene quantification. The genus-specific primer sequences used were listed in **Table S1** (see **Supplemental Method 1.4** for detail).

### Statistical Analysis

Both SPSS (20.0, SPSS Inc., Chicago, IL, USA) and R software (ver. 3.1.0, the R Project for Statistical Computing) were used for statistical analysis. In the descriptive analyses, the distribution of the data is tested by Shapiro-Wilk test before analysis. All of the characteristics except for gestational weeks of birth, TBA, and CG were in accordance with the normal distribution. Therefore, the comparisons were mainly performed with Student’s t-test, while comparisons of gestational weeks of birth, TBA, and CG between the two groups were with the Mann-Whitney rank test.

The comparisons of the relative abundance of the genera-detection using real-time PCR were performed using the t-test. The associations between genera with a prevalence ≥10% and clinical parameters were evaluated using GLM analysis, employing negative binomials depending on the distribution of the target variable using the R package ‘‘glmmADMB.’’ Before the GLM analysis, our OTU data is normalized, which means that the analysis module homogenizes the OTU sequence numbers of the samples in the OTU table, so that the sum of the OTU sequence numbers of each sample is consistent. Each sample is simply scaled up and down according to the relative abundance, that is, the shape of data distribution is not changed. Correlations between genera and clinical parameters in ICP patients were calculated using Spearman’s rank-correlation analysis with the R package ‘‘cor.test.’’

## Results

### Characteristics of the ICP and the Healthy Control Groups

There was no significant difference in age and height between the ICP and control groups **(** **Table 1** **)**. The median of TBA in ICP group was significantly higher than that in the control group (21.0 *vs.* 2.0 μmol/L, *p *< 0.01). The median of CG in the ICP group was also significantly higher than that in the control group (15.76 *vs.* 2.53 μmol/L, *p *< 0.01). Gestational weeks of birth of ICP group were earlier than that of the healthy group (37 weeks *vs.* 39 weeks, respectively, *p* < 0.01). Correspondingly, weight that increased during pregnancy (11.43 ± 3.04 kg *vs.* 13.8 ± 2.86 kg, *p* < 0.01) and BMI at delivery (24.9 ± 2.7 kg/m^2^ *vs.* 26.7 ± 2.4 kg/m^2^, *p* < 0.01) of ICP patients were less than those of the healthy controls.

### Gut Microbiota Present in the ICP and the Healthy Groups

**Figure 1:**
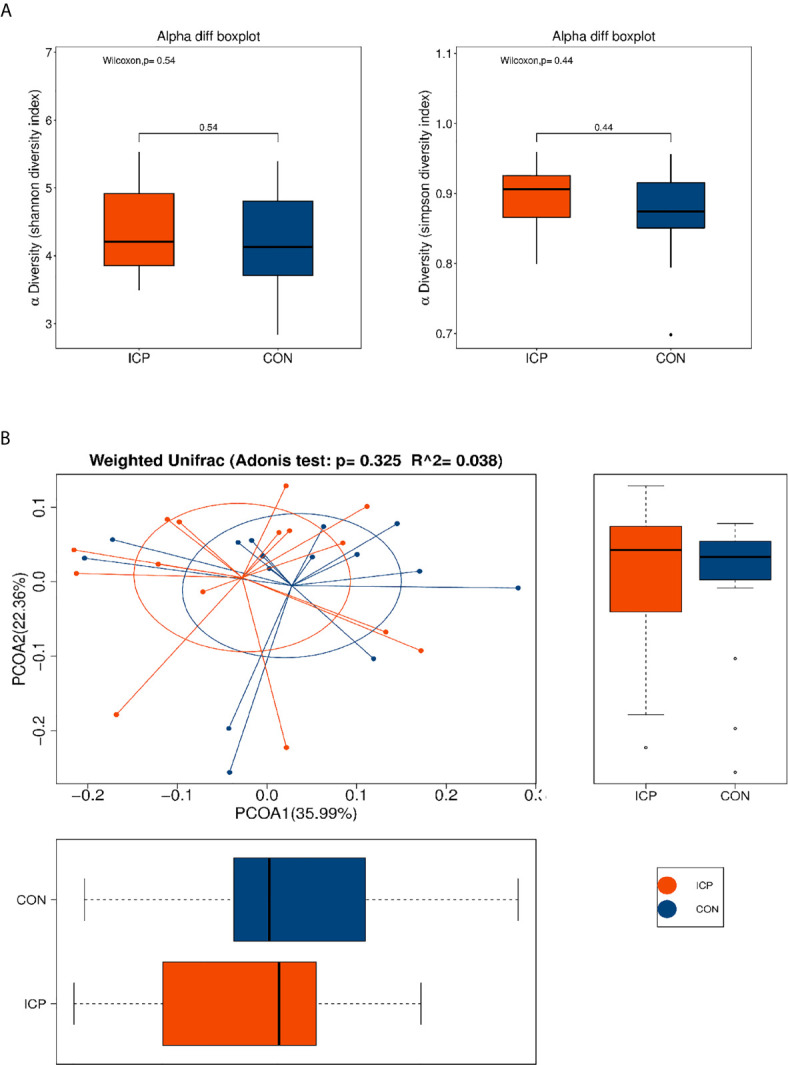
α and β-diversity of fecal microbiota in patients with ICP and healthy control (CON) subjects. **(A)** Shannon and Simpson indices were used for α-diversity, and **(B)** Adonis analysis and a principle of coordinates analysis (PCoA) indices were used for β-diversity. Each box plot represents the median, interquartile range, minimum, and maximum values. No differences were detected either in the α-diversity or in the β-diversity indices of the gut microbiota between the two groups. Red represents the ICP group, and blue represents the healthy controls (CON).

**Figure 2:**
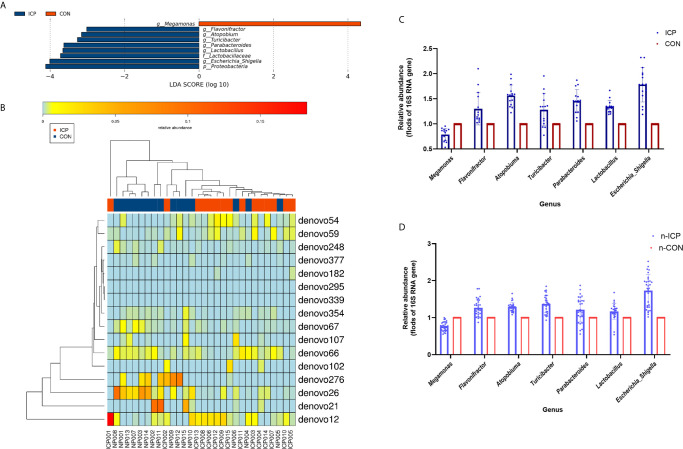
Comparison of gut microbiota between the ICP patients and healthy individuals. **(A)** Taxonomic differences in the microbial 16S rRNA gene through LEfSe analysis. **(B)** The Heatmap of differential OTUs between the ICP patients and healthy individuals. The transverse clustering shows that the abundance of the phylotypes is similar in each sample, and the longitudinal clustering shows the similarity of the expression of all phylotypes among different samples, the closer the distance is, the shorter the branch length is. Blue represents the ICP group, and red represents the healthy group. **(C)** The relative abundance of the seven genera between the ICP and healthy controls were confirmed using real-time PCR. **(D)** The relative abundances of the same seven genera were validated in the other two independent groups of 30 ICP patients and 30 healthy individuals by real-time PCR. Blue scatter plot with error bars represents the ICP patients, and red scatter plot with error bars represents the healthy controls. Light blue and light red ones represent the new 30 ICP patients and 30 healthy controls, respectively.

The profiles and diversity of the microbiota DNA were analyzed by performing high-throughput 16S rRNA gene sequencing (**Figure S1**). The species accumulation curves suggested that the sampling sufficiency and species richness are satisfactory (**Figure S2A** **)**. The total number of OTU was 431, including 384 OTUs in the ICP group and 391 OTUs in the control group (**Figure S2B**). No differences were detected either in the α-diversity or in the β-diversity indices of the gut microbiota between the two groups (**Figure 1**), suggesting that no difference was detected in the structure and richness between the ICP pregnancy and healthy controls in the third trimester. The major taxa and *p* values were listed in **Table S2**. Within LEfSe analysis, greater proportion of the genus *Megamonas* was detected in the healthy group than in the ICP group. Higher proportions of the genera *Flavonifractor*, *Atopobium*, *Turicibacter*, *Parabacteroides*, *Lactobacillus*, and *Escherichia_Shigella* were observed in the ICP patients [LDA Score (log10) >2, **Figures 2A**, **S3**). A total of 16 OTUs, 7 genera and 9 taxa with differential abundances between the ICP and healthy groups were identified (**Figures 2A, B**). The relative abundances of the mentioned taxa between the two groups were tested with the Mann-Whitney rank test (**Table 2**).

**Table 2:** The relative abundances of the differential taxa between the ICP and healthy groups.

Taxon name	Mean (CON)	Mean (ICP)	*p* Value	FDR
denovo182	0	2.45E-04	0.04	0.8
denovo377	4.84E-04	1.03E-04	0.02	0.8
denovo276	0.02	3.04E-03	0.01	0.8
denovo67	2.38E-03	5.87E-04	0.03	0.8
denovo66	4.49E-03	2.90E-03	0.03	0.8
denovo339	0	1.99E-05	0.04	0.8
denovo26	0.02	1.04E-03	8.82E-04	0.38
denovo21	0.02	2.14E-04	0.02	0.8
denovo59	1.07E-03	2.98E-03	7.45E-03	0.8
denovo54	1.05E-03	3.39E-03	0.03	0.8
denovo354	1.37E-03	4.39E-04	0.02	0.8
denovo295	2.85E-06	2.85E-05	0.01	0.8
denovo12	3.80E-03	0.02	0.02	0.8
denovo248	1.21E-03	3.56E-04	0.05	0.8
denovo107	2.61E-03	9.97E-05	0.05	0.8
denovo102	0	2.38E-03	0.04	0.8
*f:Lactobacillaceae*	0	6.95E-04	0.04	0.74
*g:Atopobium*	2.85E-06	2.85E-05	0.01	0.66
*g:Escherichia/Shigella*	3.80E-03	0.02	0.02	0.66
*g:Flavonifractor*	1.07E-03	2.98E-03	7.45E-03	0.66
*g:Lactobacillus*	0	6.95E-04	0.04	0.74
*g:Megamonas*	0.05	7.25E-03	0.02	0.66
*g:Parabacteroides*	0.01	0.02	0.03	0.66
*g:Turicibacter*	0.0001628	0.0006156	0.04	0.7
p:Proteobacteria	0.02	0.05	0.02	0.66

To determine the contribution of group factors and other confounders to significantly different genera, we performed GLM analysis of 30 individuals (Characteristics of the 30 subjects were given in **Table S3**) based on the groups factors (ICP and healthy group) and the other possible confounding factors including epidemiological factors (age, weight gain, height, gestational weeks of birth, and BMI) and other biochemical indicators (albumin, alanine aminotransferase, total bilirubin, direct bilirubin, indirect bilirubin, triglyceride, and high-density lipoprotein). The differences in the gut microbiota between the ICP patients and healthy controls were associated with the genera *Parabacteroides*, *Flavonifractor*, and *Megamonas* according to the GLM model (*p* < 0.05, **Table 3**).

**Table 3:** GLMs for the significantly different genera controlling for other confounders between the ICP and healthy groups.

Genus	Intercept	b value^†^	95% CI^‡^	*p* value
*Parabacteroides*	4.303	0.735	(0.259, 1.211)	0.011
*Flavonifractor*	1.165	1.571	(0.686, 2.455)	0.003
*Megamonas*	5.001	−5.199	(−8.612, −1.786)	0.012

^†^The b value positive (negative) mean the genus was associated with the ICP (healthy) group. ^‡^CI, confidence interval.

We also designed the genus-specific primers and performed real-time PCR to examine the relative abundance of those different genera between the ICP and healthy controls. Higher relative abundances of the genera *Flavonifractor*, *Atopobium*, *Turicibacter*, *Parabacteroides*, *Lactobacillus*, and *Escherichia_Shigella*, and lower relative abundance of the genus *Megamonas* were detected in ICP women through real-time PCR (*p*<0.05, **Figure 2C**), which was consistent with the results of the LEfSe analysis. Furthermore, we tested these genera in the gut of another independent groups of 30 ICP patients and 30 non-ICP women to validate the above results (Characteristics of the 60 subjects were given in **Table S4**), and the same alterations were detected (*p*<0.05, **Figure 2D**).

### Relationship Between Severity of ICP and Microbiota

**Figure 3:**
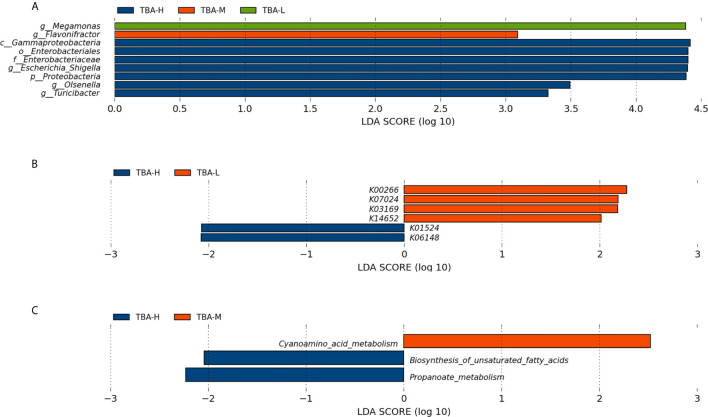
Relationship between severity of ICP and microbiota. **(A)** Taxonomic differences in the microbial 16S rRNA gene through LEfSe analysis. **(B)** Significantly different KOs were detected in the microbiome of the mild and severe ICP groups. **(C)** The microbial gene functions related to the severity of ICP were detected in the level 3 KEGG pathways. KO, KEGG (Kyoto Encyclopedia of Genes and Genomes) orthologs; Blue, red, and green squares represent the severe ICP, mild ICP, and healthy controls, respectively.

As we know, the risks of fetal complications are related to the severity of ICP patients. To better evaluate the relationship between severity of ICP and microbiota, we further divided the patients into three groups, including TBA-L (serum TBA<10 μmol/L), TBA-M (serum TBA 10–39 μmol/L), and TBA-H (serum TBA exceed 40 mol/L), that was, the healthy control group, the mild, and the severe ICP group, respectively. Within LEfSe analysis, we found that greater proportion of the genus *Megamonas* was detected in the TBA-L group. Higher proportion of the genus *Flavonifractor* was detected in the TBA-M group, and higher proportion of the genera *Escherichia_Shigella*, *Olsenella*, and *Turicibacter* were observed in the TBA-H group [LDA Score (log10) >2, **Figure 3A**].

Furthermore, PICRUSt based on *de novo* OTU was used to predict the abundances of functional categories KO in mild and severe ICP groups. In all, six significantly different KOs were detected in the microbiome of the two groups (*p* < 0.05, **Figure 3B**). In the level 3 KEGG pathways, the microbial gene function related to cyanoamino acid metabolism was increased in the mild ICP patients, whereas biosynthesis of unsaturated fatty acids and propanoate metabolism were increased in severe ICP patients (*p* < 0.05, **Figure 3C**).

### Clinical Association Between the Microbiota and ICP Characteristics

**Figure 4:**
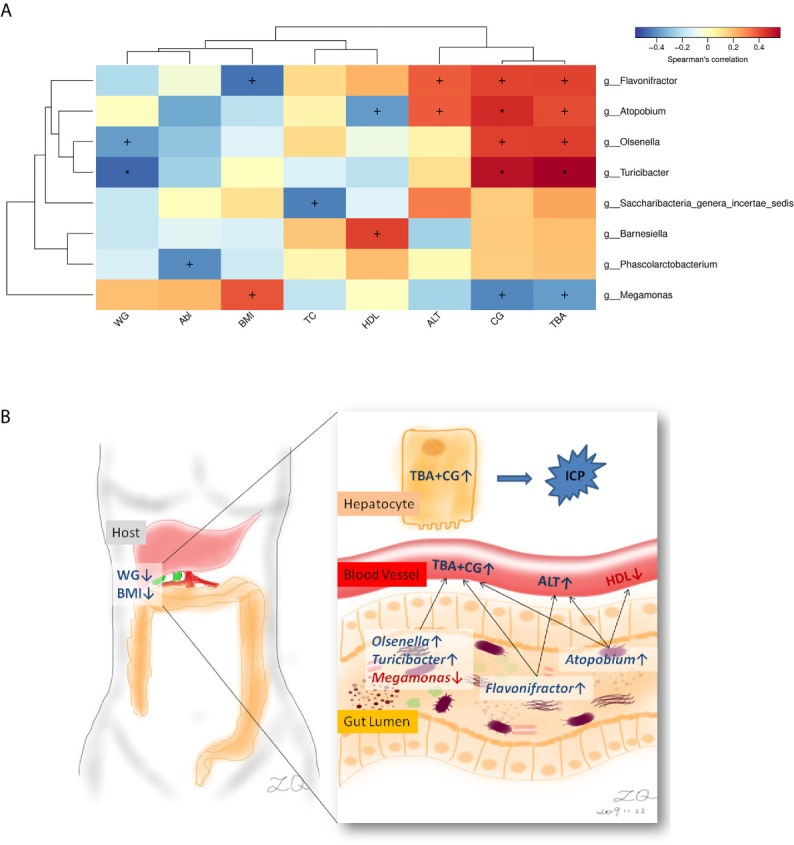
Correlations between the genera and ICP clinical characteristics. **(A)** Heatmap of correlations. **(B)** Schematic diagram showing the main genera of the gut microbes that had a predicted ICP association. Enriched genera and higher chemical indicators were shown in blue, depleted genus and lower chemical and clinical indicators were shown in red. WG, Weight that gained during pregnancy; BMI, body mass index; Alb, albumin; TC, total cholesterol; HDL, high-density lipoprotein; ALT, alanine aminotransferase; CG, cholyglycine; and TBA, total bile acid. ^+^ *p* < 0.05, *p < 0.01.

Correlation between the microbiota (genus level) and eight ICP clinical parameters was analyzed. Weight that gained during pregnancy (WG), BMI, high-density lipoprotein (HDL), alanine aminotransferase (ALT), and ICP severity (CG and TBA) were relevant to the microbiota, and the other two biochemical indicators (Alb, albumin; and TB, total bilirubin) were not relative to the microbiota that referred by LEfSe analysis (**Figure 4A**). Genus *Atopobium* and *Turicibacter* showed the most positive correlation with ICP severity indices CG and TBA (*p* < 0.01), meanwhile, Genera *Flavonifractor* and *Olsenella* showed positive correlation and *Megamonas* showed negative correlation with CG and TBA (*p* < 0.05). Besides, Genera *Atopobium* and *Flavonifractor* also showed positive correlation with ALT (*p* < 0.05). As for the other chemical indicators, Genus *Atopobium* showed negative correlation with HDL. *Turicibacter* presented the most negative correlation with WG (*p* < 0.01). *Flavonifractor* and *Megamonas* with BMI showed exactly the opposite correlation (*p* < 0.05).

## Discussion

In the present study, we observed associations between gut microbiota and ICP. Specifically, a total of seven genera and nine taxa with differential abundances between the ICP and healthy individuals were identified. The seven genera included *Escherichia_Shigella*, *Parabacteroides*, *Flavonifractor*, *Atopobium*, *Turicibacter*, *Lactobacillus*, and *Megamonas*. In addition, the family *Lactobacillaceae* and the phylum Proteobacteria were also different between the two groups. Except that the genus *Megamonas* was increased in the gut of healthy controls, the other taxa at different levels were significantly increased in the gut of the ICP group. Furthermore, through the GLM model, three key genus *Parabacteroides*, *Flavonifractor*, and *Megamonas* were confirmed after eliminating the interference factors. The genera affecting ICP patients were closely related to total bile acid and cholyglycine, as well as other biochemical indicators such as abnormally increased liver enzyme alanine aminotransferase and decreased high-density lipoprotein, and clinical variables such as weight that gained during pregnancy and body mass index (Schematic diagram see **Figure 4B**).

Weight gain during pregnancy and BMI of ICP group was significantly lower than that of the control group, which not only might be related to the timing of admission for delivery, but also might be related to excessive weight control. However, there is no definite evidence that low weight gain during pregnancy and BMI are related to ICP. Ma et al. found that participants with excessive weight gain during pregnancy had significantly higher abundance of *Atopobium* spp. as well as lower abundance of *Lactobacillus rhamnosus* in the third trimester (Ma et al., 2018), which suggested that taking probiotics may reduce the enrichment of *Atopobium* in the gut to improve the ICP. Abundance of *Megamonas* in the ICP patients was reduced, while that of the healthy controls was relatively high, which might be associated with the T cell aberration by metabolite alteration (Shimizu et al., 2019).

Genus *Lactobacillus* and *Flavonifractor* were elevated in ICP patients. Toscano et al. observed a reduction of *Flavononifractor* in healthy volunteers after 1 month of probiotic oral intake *Lactobacillus kefiri* LKF01 (DSM32079) (Toscano et al., 2017). Tedesco et al. proposed a possible mechanism by which *Lactobacillus* might improve cholestatic liver disease (Tedesco et al., 2018), so increased abundance of *Lactobacillus* might be a way of self-regulation. Nevertheless, it may also exacerbate the occurrence of liver diseases, such as alcohol-dependent and non-dependent liver disease, cirrhosis, and primary sclerosing cholangitis (Sabino et al., 2016; Dubinkina et al., 2017; Özkul et al., 2017). Therefore, probiotics supplementation during pregnancy might be a new direction to explore for the treatment of ICP, but it was still not clear whether the rise of *Lactobacillus* in the gut is a “good” self-regulation or a “bad” disease driving force, and the application of *Lactobacillus* probiotics during pregnancy still needs to be considered with caution.

In the gut of the severe ICP patients, the genera *Escherichia_Shigella* and *Turicibacter* were enriched, and the microbial gene function related to biosynthesis of unsaturated fatty acids was also increased. *Escherichia-shigella* was reported to be associated with lipid metabolism and liver enzymes, and was positively correlated with low-density lipoprotein and alanine aminotransferase (Ren S. M. et al., 2019). As for the treatments, after symbiotic treatment containing five probiotics (*Bifidobacterium longum*, *Lactobacillus acidophilus*, *Lactobacillus plantarum*, *Bifidobacterium lactis*, and *Bifidobacterium infantis*) and one prebiotic (xylooligosaccharide) for 1 and 3 months, the abundance of *Escherichia_Shigella* was significantly reduced (Huang et al., 2018); and vitamin D3 supplementation may also change the gut microbiome in the upper gastrointestinal tract with a decreased relative abundance of *Escherichia_Shigella* spp. and increased bacterial richness (Bashir et al., 2016).

Hepatic farnesoid X receptor (FXR) is expressed in liver cells and maintains the enterohepatic circulation of bile acids/salts, and high levels of bile acids in ICP patients are likely to indicate changes in FXR. Ovadia et al. found that cholic acid supplementation in mice could induce intestinal FXR signaling, which was not abrogated by pregnancy (Ovadia et al., 2019a). They also identified that UDCA-treatment in cholestatic pregnancy can alter the composition of gut microbiota and increase the proportion of *Bacteroidetes* (Ovadia et al., 2020). In our study, several genera were found to be significantly associated with the ICP severity; however, whether these differential microbiota changes after UCDA-treatment and whether they play a role through FXR signaling remains unclear. Therefore, enlargement of the population size to reduce the admission rate bias and performing unsupervised learning to stratify ICP patients into subtypes based on gut microbiota and research of underlying mechanism are recommended in future research. Microbial therapy based on microbiota subtypes might improve the outcome of traditional ICP treatment.

## Conclusions

Overall, our study demonstrated an association between the gut microbiota dysbiosis and ICP. Our findings contributed to the better understanding of the occurrence of ICP and provided a new direction to explore the microbiota subtypes and treatments for ICP.

## Data Availability Statement

We have uploaded the data to the NCBI database, and our SRA records is accessible with the following link: https://www.ncbi.nlm.nih.gov/sra/PRJNA684451.

## Ethics Statement

This studies involving human participants were reviewed and approved by the Research Ethics Committee of Women’s Hospital, Zhejiang University School of Medicine, China. The patients/participants provided their written informed consent to participate in this study.

## Author Contributions

QZ, XQ, and QL designed the experiments. QZ and XQ were responsible for the main experiments, acquisition, and analysis of data. YC and YW were responsible for collection of human samples. The data analysis of 16s rRNA and the real-time PCR were mainly performed by RW and FX. Statistics graphs were finished by RW. The date analysis of GLM was carried by WH, and the heatmaps of relationship were performed by BZ and assisted by Realbio Genomics Institute. Pictures drawn in the manuscript were finished by QZ and XQ. Manuscript was written by QZ and modified by XQ and QL. QL was also responsible for the whole study supervision. All authors contributed to the article and approved the submitted version.

## Funding

This work was supported by the National Natural Science Foundation of China (81702974), the National Key Research and Development Program of China (2018YFC1004900), and the Zhejiang Provincial Natural Science Program of China (LQ20H040008, LY20H040009).

## Conflict of Interest

The authors declare that the research was conducted in the absence of any commercial or financial relationships that could be construed as a potential conflict of interest.

## Abbreviation

16S rRNA, 16S ribosomal RNA; Alb, albumin; ALT, alanine aminotransferase; BMI, body mass index; bp, base pairs; CG, cholyglycine; GLM, generalized linear model; GWofBirth, gestational weeks of birth; HDL, high-density lipoprotein; ICP, intrahepatic cholestasis of pregnancy; KEGG, Kyoto Encyclopedia of Genes and Genomes; KO, KEGG orthologs; LCA, lithocholic acid; LDA, linear discriminant analysis; LEfSe, linear discriminant analysis effect size; OTUs, operational taxonomic units; PCR, polymerase chain reaction; PICRUSt, Phylogenetic Investigation of Communities by Reconstruction of Unobserved States; RDP, Ribosomal Database Project; SAMe, S-adenosylmethionine; SD, standard deviation; TB, total bilirubin; TBA, total bile acid; TC, total cholesterol; UDCA, ursodeoxycholic acid; WeightGain/WG, weight that gained during pregnancy.
