# Supplementary material for: Alterations of the Human Gut Microbiota in Intrahepatic Cholestasis of Pregnancy
Source: Front Cell Infect Microbiol. 2021 Apr 30;11:635680. doi: 10.3389/fcimb.2021.635680 (PMC8120235; doi:10.3389/fcimb.2021.635680)
Supplement: Supplementary file 1 [file DataSheet_1.pdf]

1. Supplemental table

Table S1 Genus-specific primer sequences for real-time PCR

Genus	Primer(5′-3′)	Amplicon size (bp†)
Megamonas	F: GGGTGCTAATACCGAATGAATG	186
	R: CGTGTCTCAGTCCCAATGTG	
Flavonifractor	F: GGGAATAACACTCCGAAAGG	292
	R: AGTTTACAACCCGAAAGCC	
Atopobium	F: ATAAAGTGGCGAACGGCTG	194
	R: CCCATAACTGCCTTGGTAGG	
Turicibacter	F: CAGCAGTAGGGAATCTTCG	264
	R: GTGGGCTTTCACATCAGAC	
Parabacteroides	F: GCGTTCCATTAGGCAGTTG	108
	R: AGGAGTTTGGTCCGTGTCTC	
Lactobacillus	F: CCCTAAAGACTGGGATACCAC	163
	R: TACGCATCATTGCCTTGG	
Escherichia_Shigella	F: ATGTCTGGGAAACTGCCTG	197
	R: TGTTGCTGGTCATCCTCTC	
16S	F: GCTCGTGTCGTGAGATGTT	159
	R: TGTAGCCCAGGTCATAAGG	

†bp, base pairs.

Table S2 List of major Taxas

Taxon name	Relative Abundance(mean)		*p* value
	ICP	CON	
**PHYLUM**			
p__Firmicutes	0.534193	0.461526	0.136974
p__Bacteroidetes	0.432855	0.475597	0.412376
p__Proteobacteria	0.022572	0.047709	0.020902
p__Actinobacteria	0.00477	0.008395	0.493634
p__Fusobacteria	0.00303	0.00334	0.923504
p__Verrucomicrobia	0.001148	0.002845	0.622857
p__Synergistetes	0.000231	0.000222	0.92689
p__Candidatus Saccharibacteria	0.000105	0.000111	0.418593
p__Euryarchaeota	0.000111	2.28E-05	1
**CLASS**			
c__Bacteroidia	0.432715	0.475557	0.412376
c__Clostridia	0.458622	0.399675	0.267097
c__Negativicutes	0.071146	0.056941	0.566774
c__Gammaproteobacteria	0.00825	0.022489	0.213323
c__Betaproteobacteria	0.010437	0.018303	0.085154
c__Actinobacteria	0.00477	0.008395	0.493634
c__Deltaproteobacteria	0.003782	0.006909	0.101266
c__Fusobacteriia	0.00303	0.00334	0.923504
c__Bacilli	0.001902	0.002939	0.220896
c__Verrucomicrobiae	0.001148	0.002845	0.622857
c__Erysipelotrichia	0.00045	0.000518	0.123474
c__Synergistia	0.000231	0.000222	0.92689
c__Methanobacteria	0.000111	2.28E-05	1
c__Alphaproteobacteria	8.26E-05	0	0.350648
c__Epsilonproteobacteria	1.99E-05	8.54E-06	0.94403
**ORDER**			
o__Bacteroidales	0.432715	0.475557	0.412376
o__Clostridiales	0.458596	0.399661	0.267097
o__Selenomonadales	0.071146	0.056941	0.566774
o__Burkholderiales	0.010437	0.018303	0.085154
o__Enterobacteriales	0.006134	0.021843	0.105584
o__Bifidobacteriales	0.003648	0.006433	0.604086
o__Desulfovibrionales	0.003229	0.006781	0.05907
o__Fusobacteriales	0.00303	0.00334	0.923504
o__Lactobacillales	0.001902	0.002939	0.220896

o__Verrucomicrobiales	0.001148	0.002845	0.622857
o__Coriobacteriales	0.000988	0.001871	0.135034
o__Pasteurellales	0.001971	0.000646	0.14908
o__Erysipelotrichales	0.00045	0.000518	0.123474
o__Synergistales	0.000231	0.000222	0.92689
o__Actinomycetales	0.000134	9.11E-05	0.208202
o__Methanobacteriales	0.000111	2.28E-05	1
o__Pseudomonadales	0.000128	0	0.350648
o__Campylobacterales	1.99E-05	8.54E-06	0.94403
o__Aeromonadales	1.71E-05	0	0.350648
**FAMILY**			
f__Bacteroidaceae	0.301369785	0.336710807	0.712965125
f__Ruminococcaceae	0.23335042	0.1993564	0.566774495
f__Lachnospiraceae	0.199282358	0.192160046	0.436289428
f__Prevotellaceae	0.097089563	0.085100384	1
f__Veillonellaceae	0.059407661	0.03556315	0.589572968
f__Porphyromonadaceae	0.023152499	0.033264986	0.1736089
f__Acidaminococcaceae	0.011738573	0.021378328	0.124818406
f__Rikenellaceae	0.008865157	0.019814894	0.41856559
f__Enterobacteriaceae	0.006134131	0.021842517	0.105583987
f__Sutterellaceae	0.008036452	0.012504628	0.589572968
f__Bifidobacteriaceae	0.003648014	0.006433148	0.604086073
f__Desulfovibrionaceae	0.003217998	0.00670084	0.06486538
f__Fusobacteriaceae	0.003030044	0.003340453	0.923503741
f__Verrucomicrobiaceae	0.001147658	0.002844938	0.62285707
f__Streptococcaceae	0.001842517	0.002050406	0.262540596
f__Coriobacteriaceae	0.000988182	0.001870995	0.135034276
f__Pasteurellaceae	0.001970668	0.000646447	0.149080477
f__Clostridiaceae 1	0.000427168	0.001156201	0.879546416
f__Peptostreptococcaceae	0.000654991	0.000726185	0.360693796
f__Erysipelotrichaceae	0.00044995	0.000518297	0.12347429
**GENUS**			
g__Bacteroides	0.30137	0.336711	0.712965
g__Faecalibacterium	0.155085	0.102925	0.074204
g__Prevotella	0.092812	0.072473	0.721791
g__Roseburia	0.047242	0.075159	0.755709
g__Lachnospiracea_incertae_sedis	0.045001	0.032001	0.309474
g__Megamonas	0.050708	0.00725	0.022789
g__Clostridium XlVa	0.025459	0.03228	0.249556
g__Gemmiger	0.02788	0.022395	0.460959
g__Phascolarctobacterium	0.011126	0.021378	0.081461
g__Parabacteroides	0.010659	0.019314	0.025087

g__Alistipes	0.008865	0.019815	0.418566
g__Dialister	0.005283	0.020225	0.499455
g__Escherichia/Shigella	0.003796	0.021401	0.022351
g__Ruminococcus	0.00653	0.012097	0.269822
g__Coprococcus	0.011291	0.004556	0.480487
g__Paraprevotella	0.003004	0.009645	0.609783
g__Parasutterella	0.00772	0.004519	0.416693
g__Ruminococcus2	0.00567	0.005362	0.678269
g__Blautia	0.005223	0.00563	0.533738
g__Bifidobacterium	0.003648	0.006433	0.604086

Table S3 Characteristics of the 30 subjects used for GLM analysis

Characteristics		ICP (n=15)	CON (n=15)	*p* Value
Pre-pregnancy	Age(years)	29.0 ± 4.5	31.3 ± 5.0	0.189
	Height(m)	1.60 ± 0.04	1.61 ± 0.05	0.585
At delivery	WeightGain‡(kg)	11.4 ± 3.0	13.8 ± 2.9	**0.037**
	Weight(kg)	64.5 ± 1.6.3	69.1 ± 8.7	0.103
	BMI† (kg/m2)	25.3 ± 3.6	26.7 ± 2.5	0.141
	GWofBirth*(weeks)	36.4 ± 2.0	38.9 ± 1.4	**0.001**
TBA¶(μmol/L)		21.0[13.0,35.0]	3.0[2.0,4.0]	**<0.000**
CG#(μg/dL)		994.0[582.0,1396.0]	188.0[110.0,220.0]	**<0.000**

‡WeightGain, weight that gained during pregnancy; BMI, body mass index; * GWofBirth, gestational weeks of birth; ¶TBA, total bile acid; #CG, cholyglycine.

Table S4 Characteristics of the independent 60 subjects used for real-time PCR

Characteristics		ICP (n=30)	CON (n=30)	*p* Value
Pre-pregnancy	Age(years)	30.4 ± 3.7	29.9 ± 3.8	0.560

	Height(m)	1.60 ± 0.05	1.61 ± 0.04	0.302
At delivery	WeightGain‡(kg)	11.1 ± 2.7	12.5 ± 3.2	0.076
	Weight(kg)	63.3 ± 7.6	69.4 ± 7.4	**0.003**
	BMI† (kg/m2)	24.7 ± 2.9	26.6 ± 2.3	**0.006**
	GWofBirth*(weeks)	37[37,38]	39[39,40]	**<0.000**
TBA¶(μmol/L)		21.0[15,27]	2.0[1.0,3.0]	**<0.000**
CG#(μg/dL)		906.80[639.70,1399.00]	142.00[116.50,175.90]	**<0.000**

‡WeightGain, weight that gained during pregnancy; BMI, body mass index; * GWofBirth, gestational weeks of birth; ¶TBA, total bile acid; #CG, cholylglycine.

2. Supplemental Figures

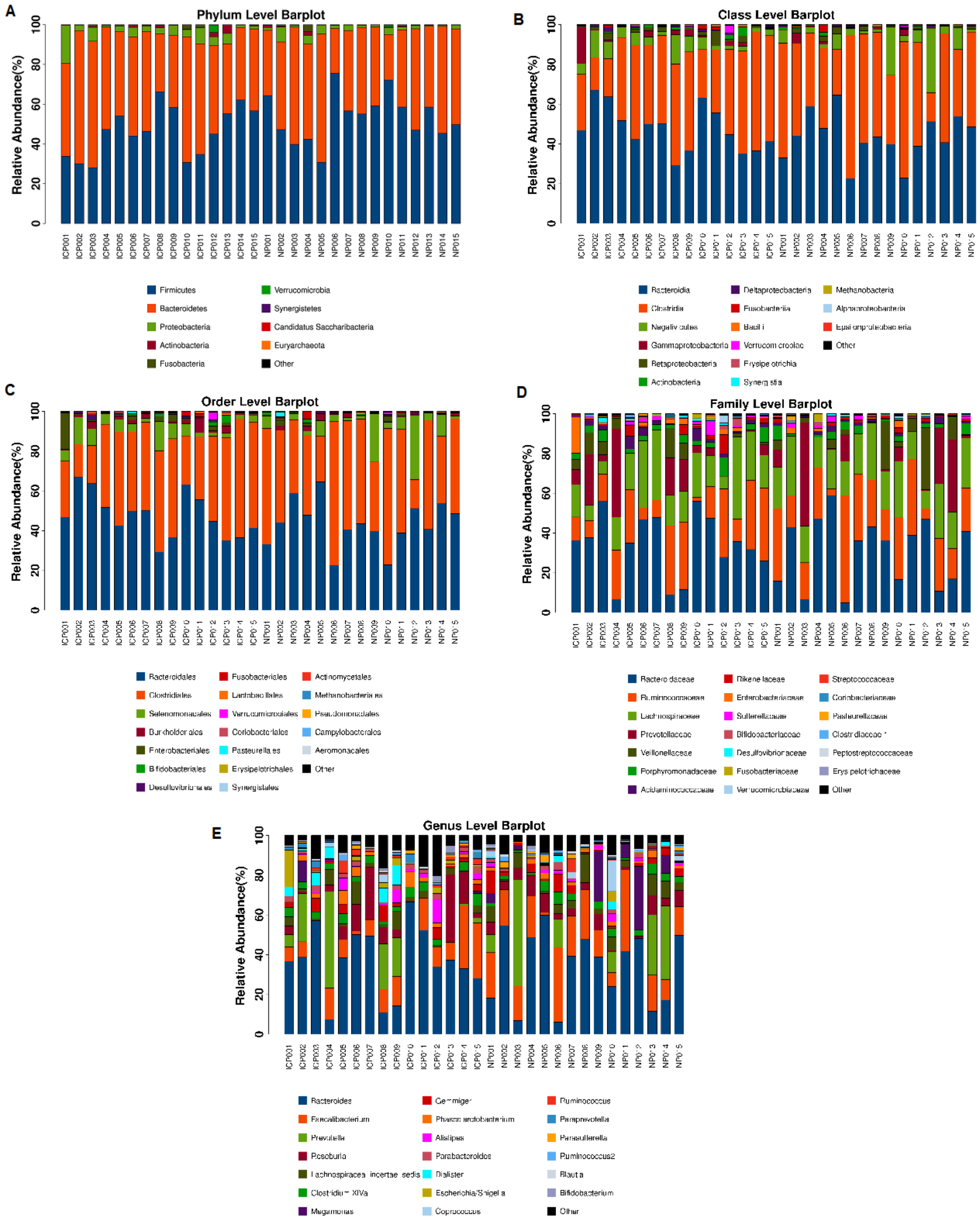

Supplementary Figure S1

The profiles and diversity of the microbiota DNA were analyzed by performing high-throughout 16S rRNA gene sequencing at the phylum (A), class (B), order (C), family (D), and genus (E) levels.

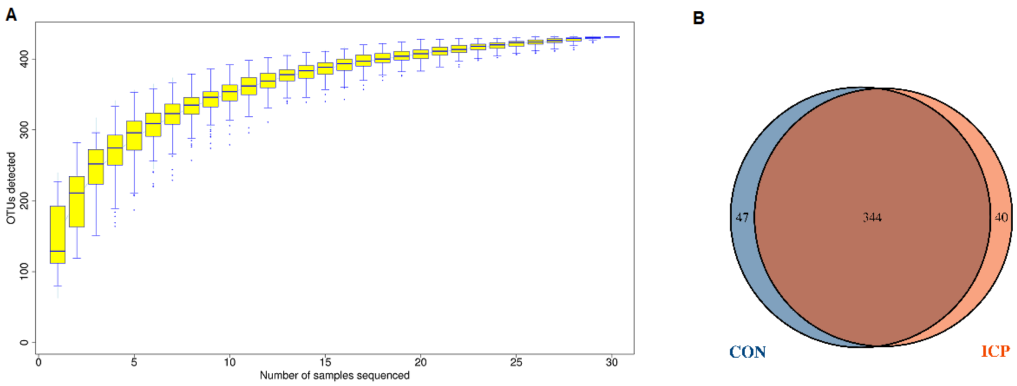

Supplementary Figure S2

The species accumulation curves (A) and the OTU profiles in the ICP and control group (B).

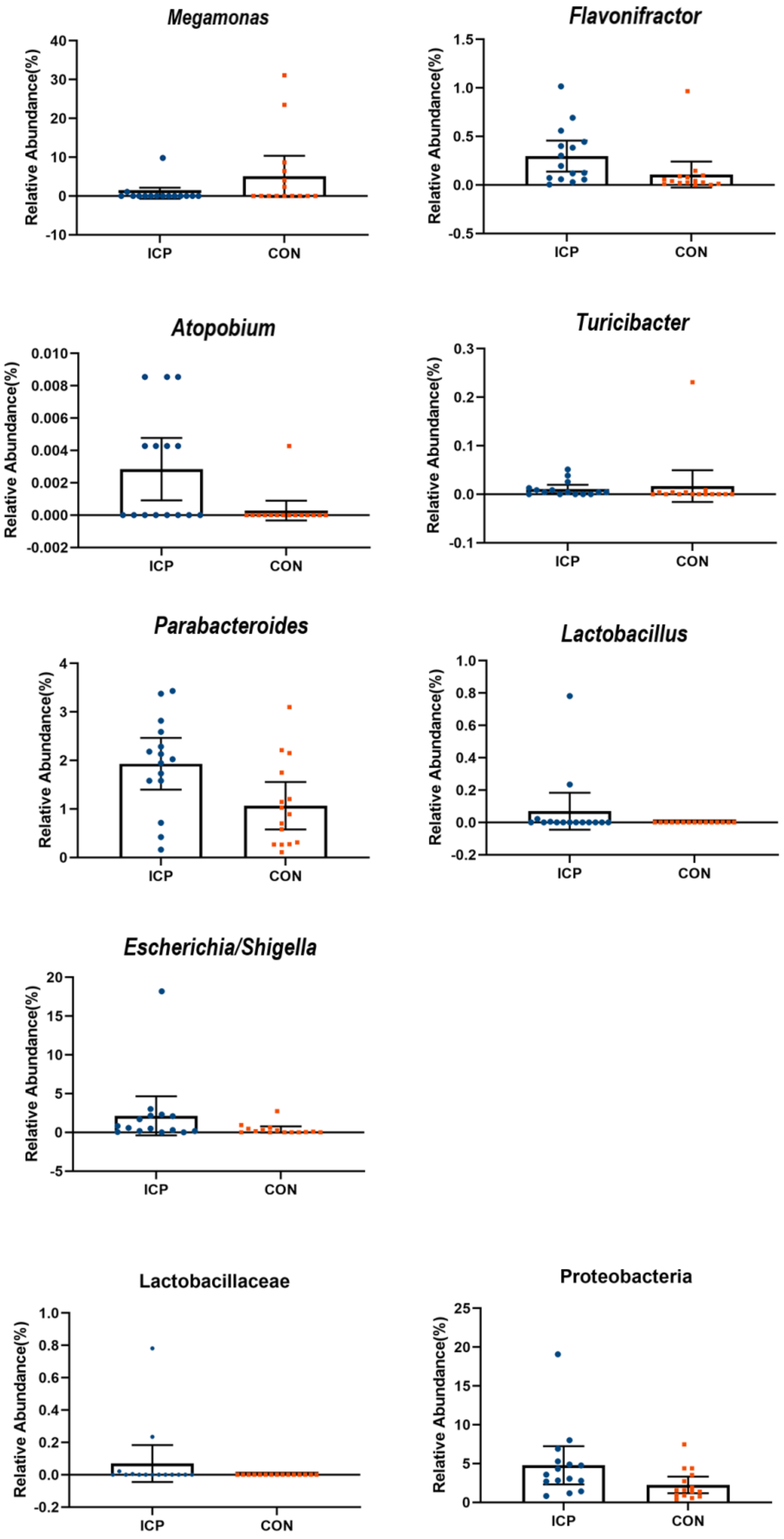

Supplementary Figure S3

A total of 7 genera (*Megamonas*, *Flavonifractor*, *Atopobium*, *Turicibacter*, *Parabacteroides*, *Lactobacillus* and *Escherichia_Shigella*) and 2 additional taxa (family *Lactobacillaceae* and phylum

Proteobacteria) with differential abundances in the ICP and healthy groups.
