# Supplementary material for: Alterations of the Human Gut Microbiota in Intrahepatic Cholestasis of Pregnancy
Source: Front Cell Infect Microbiol. 2021 Apr 30;11:635680. doi: 10.3389/fcimb.2021.635680 (PMC8120235; doi:10.3389/fcimb.2021.635680)
Supplement: Supplementary file 2 [file DataSheet_2.pdf]

Supplemental Method

1.1 DNA Extraction and PCR amplification

DNA was extracted from 180-200 mg of fecal samples using a QIAamp Fast DNA Stool Mini extraction Kit (Qiagen, Germany) according to manufacturer's protocols. The V3-V4 region of the bacteria 16S ribosomal RNA genes were amplified by PCR （95 ℃ for 3 min, followed by 30 cycles at 98℃ for 20 s, 58 ℃ for 15s, and 72 ℃ for 20 s and a final extension at 72 ℃ for 5 min) using primers 341 F 5'-CCTACGGGRSGCAGCAG)-3' and 806 R 5'-GGACTACVVGGGTATCTAATC-3'. PCR reactions were performed by using KAPA HiFi Hotstart ReadyMix PCR Kit in 30 µL mixture containing 15 µL of 2 × KAPA Library Amplification Ready Mix, 1 µL of each primer (10µM), 50ng of template DNA and ddH_2O. Extracted DNA of each sample was kept frozen at -20 ℃[1].

1.2 Illumina MiSeq PE250 sequencing

Amplicons were extracted from 2% agarose gels and purified using the AxyPrep DNA Gel Extraction Kit (Axygen Biosciences, Union City, CA, U.S.) according to the manufacturer's instructions and quantified using Qubit®2.0 (Invitrogen, U.S.). After preparation of library, these tags were sequenced on MiSeq platform (Illumina, Inc., CA, USA) by using MiSeq Reagent kit v3 (MS-102-2003) for paired end reads of 250 base pairs (bp), which were overlapped on their 3 ends for concatenation into original longer tags. DNA extraction, library construction and sequencing were conducted at

Realbio Genomics Institute (Shanghai, China).

1.3 Process of sequencing data

PANDAseq(V2.9)[2] is employed to assemble pairs of reads into complete sequences in the hypervariable region using overlapped bases. DNA sequencing libraries targeting the V3–V4 hypervariable regions of the 16S rRNA gene were prepared by PCR amplfication using specific primers supplemented with Illumina sequencing adapters and sample-specfic barcodes according to Illumina’s instructions (https://support.illumina.com/downloads/16s_metagenomic_sequencing_library_preparation. html). The V3–V4 regions of the bacterial 16S rRNA gene were amplified using the universal primers 338F 5’-barcode-ACTCCTACGGGAGGCAGCAG-3’ and 806R 5’-barcode-GGACTACHVGGGTWTCTAAT-3’, which contain a short sequence barcode unique to each sample.

Tags, trimmed of barcodes and primers, were further checked on their rest lengths and average base quality. 16S tags were restricted between 250 and 500 nt such that the average Phred score of bases was no worse than 20 (Q20) and no more than 3 ambiguous N. The copy number of tags was enumetated and redundancy of repeated tags was removed. Only the tags with frequency more than 1, which tend to be more reliable, were clustered into OTUs, each of which had a representative tag. Operational Taxonomic Units (OTUs) were clustered with 97% similarity using UPARSE[3] （http://drive5.com/uparse/) and chimeric sequences were identified and removed using Usearch (version 7.0). Each representative tag was assigned to a taxa

by Ribosomal Database Project (RDP) Classifer (http://rdp.cme.msu.edu/) against the RDP database (http://rdp.cme.msu.edu/) using confidence threshold of 0.8. OTU profling table and α-/β- diversity analyses were also achieved by python scripts of Qiime (V1.9.1) [4]. The total samples resulted in 1,077,564 clean reads with an average of 35,918.8 ±1,980.3 clean tags per sample.

The α-diversity and β-diversity indices were calculated based on the rarefied OTU counts using the Qiime program. We randomly selected 23,410 reads for each sample. The α-diversity represents an analysis of diversity in a single sample reflected by parameters including good coverage, Chao 1, whole tree, Shannon index and Simpson index using Qiime**.** Wilcoxon rank sum test was used to compare each α-diversity index. β-diversity is used as a measure of the microbiota structure between the two groups. Both the weighted and unweighted Unifrac distance matrices were plotted in the principal coordinate analysis (PCoA), and analyses of similarities (ANOSIMs) were performed using the R “vegan“ package.

For taxa with a prevalence≥10%, differential abundance analysis was performed using the Wilcoxon rank-sum test at the phylum, class, order, family, and genus levels. For multiple comparisons of bacterial counts, the false discovery rate was calculated using the Benjamini and Hochberg method. Microorganism features used to distinguish the microbiotas specific to ICP were identified using the linear discriminant analysis (LDA) effect size (LEfSe)[5] method with an alpha cutoff of 0.05 and an effect size cutoff of 2.0.

Phylogenetic Investigation of Communities by Reconstruction of Unobserved

States (PICRUSt)[6] was used to predict the abundances of functional categories in the Kyoto Encyclopedia of Genes and Genomes (KEGG) orthologs (KO). The graph of KEGG pathways in level 2 (41pathways) and level 3 (328pathways) 2 was performed with STAMP [7], and *p* values were calculated with White's non-parametric t-test.

For the PICRUSt analysis, the OTU clustering method we used is de novo OTU clustering. The analysis software used is Usearch (version 7.0), and the database used is RDP database (http://rdp.cme.msu.edu). Firstly, the singletons in the spliced long reads were filtered out, because the singletons may be caused by sequencing errors. This part of the sequence was removed without cluster analysis. Usearch was used to cluster under the similarity of 0.97, and the clustered sequences were filtered by chimerism to obtain OTUs for species classification.

1.4 Genus-specific quantification by real-time PCR

The comparison and analysis of the 16S region sequences with those of differential genera reference strain were carried out by the multiple alignment program DNAman software. The specific primer target sites for the quantitative analysis were performed using Primer 5. Universal 16S rRNA gene was used as the internal reference and the abundances of the genera were expressed as relative levels to 16S rRNA. The PCR reaction and condition were the same with 16S rRNA gene quantification（95 ℃ for 3 min, followed by 30 cycles at 98℃ for 20 s, 58 ℃ for 15s, and 72 ℃ for 20 s and a final extension at 72 ℃ for 5 min). The genus-specific primer sequences used were listed in **TableS1.** Data (three biological repeats and three technical repeats at each

time point) were analyzed by the comparative threshold cycle (CT) method and the standard formula. Each reaction was performed in triplicate.

References

1. Mirsepasi H, Persson S, Struve C, *et al*. Microbial diversity in fecal samples depends on DNA extraction method: easyMag DNA extraction compared to QIAamp DNA stool mini kit extraction. *BMC Research Notes* 2014;7:50.
2. Masella AP, Bartram AK, Truszkowski JM, *et al*. PANDAseq: paired-end assembler for illumina sequences. *BMC Bioinformatics* 2012;13:31.
3. Edgar RC. UPARSE: highly accurate OTU sequences from microbial amplicon reads. *Nature Methods* 2013;10:996.
4. Caporaso JG, Kuczynski J, Stombaugh J, *et al*. QIIME allows integration and analysis of high-throughput community sequencing data. *Nature Methods* 2010;7:335–336.
5. Segata N, Izard J, Waldron L, *et al*. Metagenomic biomarker discovery and explanation. *Genome Biol*. 2011;12:R60.
6. Langille MGI, Zaneveld J, Caporaso JG, *et al*. Predictive functional profiling of microbial communities using 16S rRNA marker gene sequences. *Nature Biotechnology.* 2013;31:814-812.
7. Parks DH, Tyson GW, Hugenholtz P, *et al*. STAMP: Statistical analysis of taxonomic and functional profiles. *Bioinformatics.* 2014;30:3123-3124.
